# Critical role of GRP receptor–expressing neurons in the spinal transmission of imiquimod‐induced psoriatic itch

**DOI:** 10.1002/npr2.12120

**Published:** 2020-06-25

**Authors:** Norikazu Kiguchi, Fumihiro Saika, Yohji Fukazawa, Shinsuke Matsuzaki, Shiroh Kishioka

**Affiliations:** ^1^ Department of Pharmacology Wakayama Medical University Wakayama Japan; ^2^ Department of Anatomy Kansai University of Health Sciences Osaka Japan; ^3^ Faculty of Wakayama Health Care Sciences Takarazuka University of Medical and Health Care Wakayama Japan

**Keywords:** AMPA, GRP, pruritus, psoriasis, spinal cord

## Abstract

**Aim:**

Ample evidence indicates that gastrin‐releasing peptide receptor (GRPR)–expressing neurons play a critical role in the transmission of acute itch. However, the pathophysiology of spinal mechanisms underlying intractable itch such as psoriasis remains unclear. In this study, we aimed to determine whether itch‐responsive GRPR^+^ neurons contribute to the spinal transmission of imiquimod (IMQ)‐induced psoriatic itch.

**Methods:**

To generate a psoriasis model, C57BL/6J mice received a daily topical application of 5% IMQ cream on their shaved back skin for 7‐10 consecutive days. GRP^+^ neurons were inhibited using Cre‐dependent expression of Gi‐designer receptors exclusively activated by designer drugs (DREADDs), while GRPR^+^ neurons were ablated by intrathecal administration of bombesin‐saporin.

**Results:**

Repeated topical application of IMQ elicited psoriasis‐like dermatitis and scratching behaviors. The mRNA expression levels of *GRP* and *GRPR* were upregulated in the cervical spinal dorsal horn (SDH) on days 7 and 10 after IMQ application. Either chemogenetic silencing of GRP^+^ neurons by Gi‐DREADD or ablation of GRPR^+^ neurons significantly attenuated IMQ‐induced scratching behaviors.

**Conclusion:**

The GRP‐GRPR system might be enhanced in the SDH, and itch‐responsive GRPR^+^ neurons largely contribute to intractable itch in a mouse model of psoriasis.

## Abstract 2

The aim of this study was to determine whether itch‐responsive gastrin‐releasing peptide receptor (GRPR)–expressing neurons contribute to the spinal transmission of imiquimod (IMQ)‐induced psoriatic itch. We found that mRNA expression of *GRP* and *GRPR* was upregulated in the cervical spinal dorsal horn after IMQ application, and IMQ‐induced psoriatic itch was suppressed by either chemogenetic inhibition of GRP^+^ neurons or ablation of GRPR^+^ neurons.

## Graphical abstract

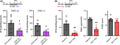

## 1 INTRODUCTION

Itch (pruritus) is an unpleasant sensation that elicits a desire or reflex to scratch. Accumulating evidence suggests that gastrin‐releasing peptide receptor (GRPR)–expressing neurons play a central role in the spinal transmission of chemically induced acute itch.^1^, ^2^ In fact, intrathecal (i.t.) delivery of GRP evokes robust scratching behaviors in not only rodents but also primates.^3^, ^4^, ^5^ Moreover, we have demonstrated that GRP and glutamate cooperatively activate GRPR^+^ neurons through GRPR and α‐amino‐3‐hydroxy‐5‐methyl‐4‐isoxazolepropionic acid receptor (AMPAR), regulating acute itch originated by sensory C‐fiber activation.^6^

Despite scientific knowledge for mechanisms of acute itch, the pathophysiology of intractable itch remains unclear. Given that intractable itch associated with various skin diseases causes serious economic loss globally because standard antipruritic agents have limited effectiveness, there is a strong need to develop novel mechanism‐based therapeutics.^1^, ^7^ Psoriasis is a chronic inflammatory skin disease that causes rapid turnover of skin cells and produces thick, red, and itchy skin covered with silvery scales.^8^, ^9^ Much attention has been focused on inflammatory mechanisms in the skin, while knowledge of spinal mechanisms underlying psoriatic itch remains limited. In this study, we aimed to determine whether itch‐responsive GRPR^+^ neurons contribute to the spinal transmission of imiquimod‐induced psoriatic itch.

## 2 METHODS

### 2.1 Mice

All animal experiments were approved by the Animal Research Committee of Wakayama Medical University and were conducted in accordance with the in‐house guidelines for the care and use of laboratory animals of Wakayama Medical University and the ARRIVE guidelines. Male C57BL/6J (6‐7 weeks old) mice were purchased from SLC (Hamamatsu, Japan). R26‐LSL‐Gi‐DREADD mice [B6N.129‐Gt(ROSA)26Sor^tm1(CAG‐CHRM4*,−mCitrine)Ute^/J; stock #026219] and GRP‐Cre (Tg) mice [B6.FVB(Cg)‐Tg(Grp‐cre)KH288Gsat/Mmucd; stock #037585] were purchased from The Jackson Laboratory and Mutant Mouse Resource & Research Centers (MMRRC), respectively. For Cre‐dependent expression of Gi‐designer receptors exclusively activated by designer drugs (DREADDs) in the Rosa locus of GRP‐expressing cells, R26‐LSL‐Gi‐DREADD mice were crossed with GRP‐Cre mice.^6^, ^10^ Subsequently, male mice heterozygous for ROSA26 and GRP‐Cre (transgenic) were used for the experiments. Mice were housed in plastic cages in a temperature‐controlled room (23°C–24°C, 60%–70% humidity) with a 12‐h dark/light cycle and provided with water and food ad libitum.

### 2.2 Psoriatic itch

After shaving fur on the rostral back with an electric clipper, mice received a daily topical application of 62.5 mg Beselna cream (5% imiquimod, IMQ; Mochida Pharmaceutical Co., Ltd.) on their shaved back skin for 7‐10 consecutive days.^11^, ^12^ The psoriasis area and severity index (PASI) for erythema, scaling, and thickness was scored independently from 0 to 4 (0, none; 1, mild; 2, moderate; 3 severe; and 4, very severe), and the total score was presented. For the evaluation of itch‐related scratching behaviors, mice were placed in plastic cages (20 × 12 × 12 cm^3^) with a small amount of bedding. The number of scratching bouts was measured in 10‐min intervals for 40 minutes as reported previously.^5^ One scratching bout was defined as lifting the hind paw to scratch the nape regions and then returning the paw to the floor or to the mouth for licking. Analyses were carried out in a blinded fashion.

### 2.3 RT‐qPCR

Mice were euthanized by decapitation, and fresh cervical (C3‐5) spinal dorsal horn (SDH) tissue was collected in RNAlater solution (Thermo Fisher Scientific). The TRIzol^®^ Plus RNA Purification Kit (Thermo Fisher Scientific) was used for the isolation of total RNA from the tissues following the manufacturer's instructions. Briefly, tissues were placed in a 1.5‐mL RNase‐free tube and homogenized with TRIzol reagent. Chloroform was added to each sample, and samples were then centrifuged at 4°C for 15 minutes. The aqueous phase containing RNA was transferred to a fresh tube, and RNA was isolated using a purification column. Total RNA extract was used for the synthesis of cDNA by reverse transcription as follows: Total RNA was incubated with Random Primers (Promega) at 70°C for 5 minutes and then was cooled on ice. Samples were converted to cDNA by incubation with M‐MLV Reverse Transcriptase and dNTPMix (Promega) at 37°C for 50 minutes. qPCR was performed using the AriaMx Real‐Time PCR System (Agilent Technologies) by using the cDNA as the template, primers for each gene and SYBR^®^ Premix Ex Taq™ II (Takara Bio). The primers for β‐actin (*ACTB*; 5′‐CAGCTGAGAGGGAAATCGTG‐3′, 5′‐TCTCCAGGGAGGAAGAGGAT‐3′), *GRP* (5′‐GGAAGAAGCTGCAAGGGATT‐3′, 5′‐GATCCCAAGTAGGCTGGAGA‐3′), and *GRPR* (5′‐CCCTGCAGTTTATGGGCTTA‐3′, 5′‐GAACAGGTTTGGCACGTTTC‐3′) were purchased from Thermo Fisher Scientific. Reactions were performed under the following conditions: 3 minutes at 95°C followed by 45 cycles of two steps (10 s at 95°C and 30 s at 60°C). The fluorescence intensities were recorded, and data were normalized to *ACTB*.

### 2.4 Drug administration

Bombesin‐saporin (Bom‐Sap; Advanced Targeting Systems) and blank‐Sap (Advanced Targeting Systems) were dissolved in sterile phosphate‐buffered saline (PBS) and were administered intrathecally (i.t.) in a volume of 5 μL as described previously.^6^ Under isoflurane anesthesia, mice were secured by a firm grip on the pelvic girdle and drugs were injected by lumbar puncture between L5 and L6 vertebrae using a 30‐gauge needle fitted with a Hamilton microsyringe. Clozapine‐N‐oxide (CNO; Enzo Life Sciences) was dissolved in physiological saline and was administered intraperitoneally (i.p.; 0.3 mg/kg) to awake mice in a volume of 0.1 mL/10 g body weight or i.t. (3 nmol) to anesthetized mice in a volume of 5 μL.

### 2.5 Data analysis

Data are presented as mean ± standard error of the mean (SEM). Statistical analyses were performed using Student's *t* test, one‐way analysis of variance followed by Tukey's multiple comparison test, or two‐way analysis of variance followed by Bonferroni's multiple comparison test, as appropriate. Statistical significance was established at *P* < .05.

## 3 RESULTS

**FIGURE 1:**
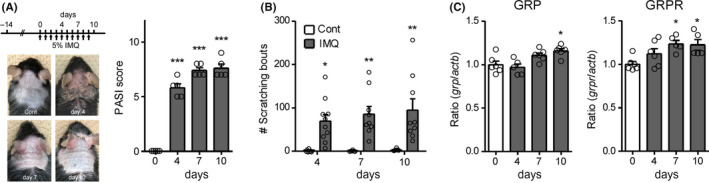
Imiquimod (IMQ)‐induced psoriasis and upregulation of *GRP* and *GRPR* in the spinal dorsal horn (SDH). A, Schedule of the IMQ application and photograph and psoriasis area and severity index (PASI) score of the IMQ‐treated mice on days 0, 4, 7, and 10. n = 5. ***P < .001 vs. control. B, Scratching bouts were observed on days 4, 7, and 10 before IMQ application up to 40 min, and the total number of scratching bouts is shown. n = 6‐10. **P < .01, *P < .05 vs. control. C, The mRNA expression levels of *GRP* and *GRPR* on days 0, 4, 7, and 10 in the cervical SDH were evaluated by RT‐qPCR. n = 5‐6. *P < .05 vs. control

**FIGURE 2:**
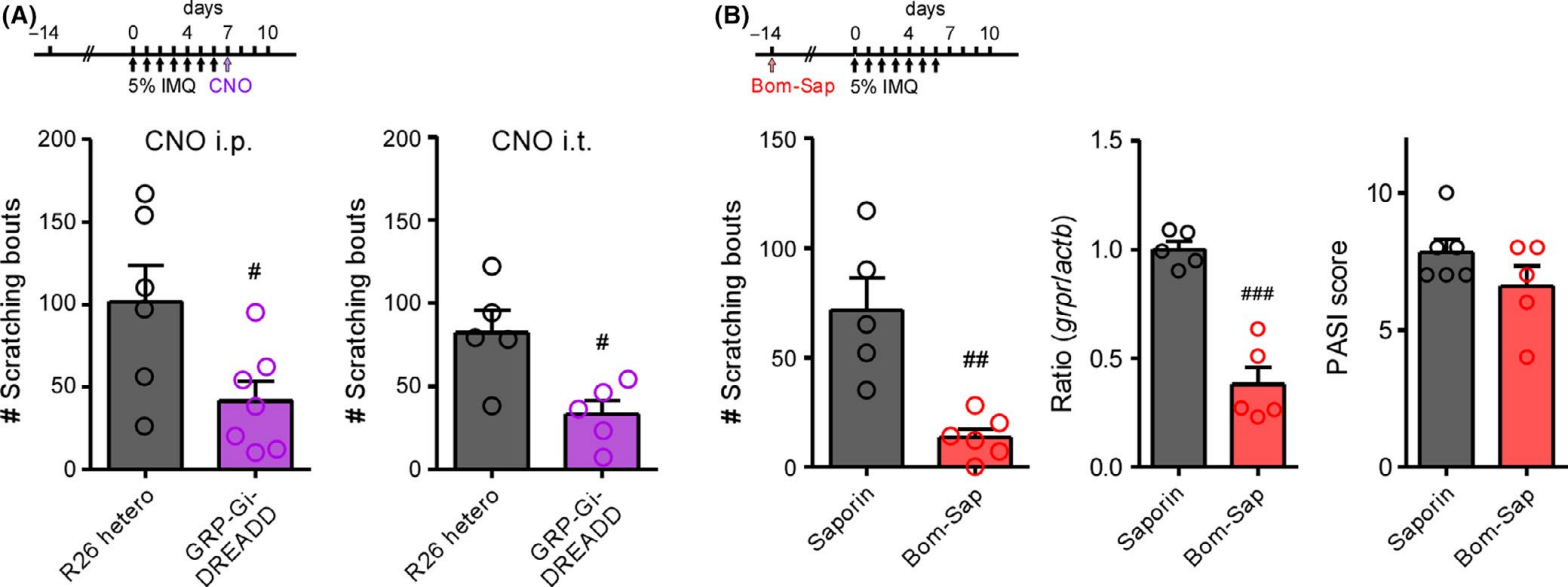
Roles of GRP^+^ and GRPR^+^ neurons in IMQ‐induced psoriatic itch. A, Inhibition of scratching behaviors by intraperitoneally (i.p.; 0.3 mg/kg; left panel) or intrathecally (i.t.; 3 nmol, right panel) administration of clozapine‐N‐oxide (CNO, 30 min prior to observation) in GRP‐Gi‐DREADD mice on day 7 after IMQ application; n = 5‐7. ^#^ *P* < .05 vs. control. B, Prevention of scratching behaviors in bombesin‐saporin (Bom‐Sap)‐treated (250 ng, i.t., 14 days before experiment) mice on day 4 after IMQ application. n = 5‐6. ^##^ *P* < .01 vs. control. The mRNA expression levels of *GRPR* in the cervical SDH and PASI score were evaluated on day 7 after IMQ application. n = 5‐6. ^###^ *P* < .001 vs. control

Repeated topical application of IMQ, a toll‐like receptor 7 agonist, increased PASI score indicating psoriasis‐like dermatitis (ie, erythema, scaling, and thickness) [F(3,16) = 140.7, *P* < .0001; Tukey's test, *P* < .001; n = 5; Figure 1A] and elicited scratching behaviors [F(1,42) = 34.43, *P* < .0001; Bonferroni's test, *P* < .01; n = 6‐10; Figure 1B). In the SDH, on days 7 and 10 after IMQ application, the mRNA expression levels of *GRP* [F(3,19) = 6.710, *P* = .0028; Tukey's test, *P* < .05; n = 5‐6] and *GRPR* [F(3,18) = 4.823, *P* = .0123; Tukey's test, *P* < .05; n = 5‐6] were significantly upregulated (Figure 1C). To determine the involvement of spinal GRP^+^ neurons in IMQ‐induced psoriatic itch, we conducted chemogenetic silencing of GRP^+^ neurons by crossing R26‐LSL‐Gi‐DREADD mice and GRP‐Cre mice. As we previously reported,^6^ 0.3 mg/kg (i.p.) or 3 nmol (i.t.) of CNO, a ligand for Gi‐DREADD, was used for selective induction of the GRP‐Gi‐DREADD. On day 7 after IMQ application, IMQ‐induced scratching behaviors were attenuated by either i.p. [t(11) = 2.491, *P* = .0300; n = 6‐7] or i.t. [t(8) = 3.069, *P* = .0154; n = 5] administration of CNO (30 minutes prior to observation) in GRP‐Gi‐DREADD mice compared to control (R26 hetero) mice (Figure 2A). Finally, we determined whether GRPR^+^ neurons are pivotal for IMQ‐induced psoriatic itch by the ablation of GRPR^+^ neurons following i.t. administration of Bom‐Sap. Compared to control (blank‐saporin), IMQ‐induced scratching behaviors were markedly suppressed [t(9) = 4.237, *P* = .0022; n = 5‐6] with Bom‐Sap treatment that significantly ablated GRPR^+^ neurons in the SDH [t(8) = 7.013, *P* < .001; n = 5]. On the other hand, Bom‐Sap treatment had no significant effect on PASI score on day 7 after IMQ application (Figure 2B).

## 4 DISCUSSION

This study provides two novel findings: mRNA expression of *GRP* and *GRPR* is upregulated in the SDH after IMQ application, and IMQ‐induced psoriatic itch was suppressed by either chemogenetic inhibition of GRP^+^ or ablation of GRPR^+^ neurons. Similar to contact dermatitis as previously reported, ^6^ upregulation of GRP and GRPR directly indicates the enhancement of the GRP‐GRPR system under psoriatic itch. Given that GRP^+^ neurons are excitatory glutamatergic neurons,^13^, ^14^ GRP and glutamate may cooperatively activate itch‐responsive GRPR^+^ neurons in the SDH under the condition of psoriatic itch.

Although spinal circuits underlying itch are largely complicated, several key neurons participating in these pathways have been determined within this decade. In particular, chemically elicited acute itch through the activation of pruriceptors in sensory C‐fibers is integrated in GRPR^+^ neurons in the SDH, which input to projection neurons to the brain.^1^, ^2^ Nevertheless, how such systems contribute to skin disease‐associated intractable itch has remained unclear. The neuro‐immune interactions within the skin involved in intractable itch are well investigated,^15^ but there is an urgent need to clarify the spinal mechanisms underlying intractable itch.

Collectively, we have demonstrated that itch‐responsive GRPR^+^ neurons largely contribute to intractable itch in a mouse model of psoriasis. Pharmacological interventions targeting ligand‐receptor systems, such as GRP‐GRPR and glutamate‐AMPAR systems, may lead to novel therapeutics for intractable itch.

## CONFLICT OF INTEREST

The authors have no conflict of interest.

## AUTHOR CONTRIBUTION

NK participated in research design. NK, FS, and YF performed experiments. NK, FS, SM, and SK analyzed data. NK wrote the manuscript.

## ANIMAL STUDIES

All animal experiments were approved by the Animal Research Committee of Wakayama Medical University.

## Supporting information

Table S1

## Data Availability

The data that supports the findings of this study are available in the Supporting information of this article.
